# Pulse Rate Variability in Emergency Physicians During Shifts: Pilot Cross-Sectional Study

**DOI:** 10.2196/13909

**Published:** 2019-10-02

**Authors:** Gregory Andrew Peters, Matthew L Wong, Joshua W Joseph, Leon D Sanchez

**Affiliations:** 1 Department of Emergency Medicine Beth Israel Deaconess Medical Center Boston, MA United States; 2 Harvard Medical School Boston, MA United States

**Keywords:** emergency medicine, burnout, photoplethysmography, emergency physicians, physician wellness, stress, heart rate variability, pulse rate variability

## Abstract

**Background:**

The high prevalence of physician burnout, particularly in emergency medicine, has garnered national attention in recent years. Objective means of measuring stress while at work can facilitate research into stress reduction interventions, and wearable photoplethysmography (PPG) technology has been proposed as a potential solution. However, the use of low-burden wearable biosensors to study training and clinical practice among emergency physicians (EP) remains untested.

**Objective:**

This pilot study aimed to (1) determine the feasibility of recording on-shift photoplethysmographic data from EP, (2) assess the quality of these data, and (3) calculate standard pulse rate variability (PRV) metrics from the acquired dataset and examine patterns in these variables over the course of an academic year.

**Methods:**

A total of 21 EP wore PPG biosensors on their wrists during clinical work in the emergency department during a 9-hour shift. Recordings were collected during the first quarter of the academic year, then again during the fourth quarter of the same year for comparison. The overall rate of usable data collection per time was computed. Standard pulse rate (PR) and PRV metrics from these two time points were calculated and entered into Student *t* tests.

**Results:**

More than 400 hours of data were entered into these analyses. Interpretable data were captured during 8.54% of the total recording time overall. In the fourth quarter of the academic year compared with the first quarter, there was no significant difference in median PR (75.8 vs 76.8; *P*=.57), mean R-R interval (0.81 vs 0.80; *P*=.32), SD of R-R interval (0.11 vs 0.11; *P*=.93), root mean square of successive difference of R-R interval (0.81 vs 0.80; *P*=.96), low-frequency power (3.5×103 vs 3.4×103; *P*=.79), high-frequency power (8.5×103 vs 8.3×103; *P*=.91), or low-frequency to high-frequency ratio (0.42 vs 0.41; *P*=.43), respectively. Power estimates for each of these tests exceeded .90. A secondary analysis of the resident-only subgroup similarly showed no significant differences over time, despite power estimates greater than .80.

**Conclusions:**

Although the use of PPG biosensors to record real-time physiological data from EP while providing clinical care seems operationally feasible, this study fails to support the notion that such an approach can efficiently provide reliable estimates of metrics of interest. No significant differences in PR or PRV metrics were found at the end of the year compared with the beginning. Although these methods may offer useful applications to other domains, it may currently have limited utility in the contexts of physician training and wellness.

## Introduction

The concept of burnout in the workplace, roughly defined as mental exhaustion because of chronic work-related stress, was first introduced nearly 50 years ago [1]. Once applied to physicians, the field of physician burnout research has steadily grown at a rapid rate [2], especially as it pertains to medical training in the United States [3]. A recent study of more than 16,000 US residents showed that the majority of their sample reported burnout [4]. In particular, emergency medicine (EM) has been identified by this body of research as a relatively high-risk specialty, as emergency physicians (EPs) frequently show the highest rates of burnout [5,6]. As physician burnout continues to increase in prevalence and attracts greater attention both within and outside medicine, efforts to alleviate burnout among medical providers have become a priority [7-10]. A natural corollary from this movement has been an internationally concerted search for new, objective ways to measure burnout, to serve as a complementary approach to the current body of research predominated by surveys and self-report inventories such as the Maslach Burnout Inventory [11].

In this search for new technologies to direct efforts to relieve physician burnout, heart rate (HR) and heart rate variability (HRV) metrics have recently shown promise in multiple applications to medical providers [12-17]. Clinical applications of HRV analysis trace roots back more than a century ago [18], and the combination of improved technology and increased interest in physician well-being have spurred this new area of investigation [19]. Despite concerns regarding the validity of certain applications of HRV data (eg, the use of low-frequency [LF] power of HRV—and in turn ratio of LF to high-frequency [HF] power—to measure sympathovagal balance has been criticized [20-23]), a large body of evidence supports the notion that HRV analysis can illuminate the balance between sympathetic and parasympathetic tone in the body [24], enabling the use of HRV to study autonomic responses to mental stress [25]. A particularly noteworthy study of HRV in surgeons highlights the value of objective measures of stress among medical providers, reporting that acute care surgeons in their sample showed levels of physiologic stress that were elevated out of proportion to self-reported stress [17]. These results offer compelling evidence that physicians, especially those routinely exposed to traumatic situations in highly time-sensitive settings, might provide unreliable assessments of their own stress. Thus, widespread use of objective, ecologically valid metrics of stress might represent a key piece of the puzzle in efforts to understand and alleviate burnout among EPs.

While HRV recorded by electrocardiography (ECG) offers a seemingly effective means by which to chart physiologic manifestations of stress responses in small samples of providers for limited durations, widespread application of this methodology to routine monitoring of physicians and trainees at work remains unfeasible. However, measurement of pulse rate (PR) and pulse rate variability (PRV) via wearable photoplethysmography (PPG) biosensor technology has been posed as an extremely low-burden alterative to ECG measurement with significantly greater potential for scalability. In a basic sense, while ECG measures changes in electrical activity in the heart, PPG measures changes in blood volume pulse (BVP) in the peripheral vasculature. The context of this physiologic relationship lies in the assumption that chronic exposures to workplace stressors that contribute to burnout are psychologically experienced by EPs, corresponding to neurological registration of the experience that triggers an autonomic response. This autonomic response, expressed as a change in sympathovagal balance, directly influences cardiac function as measurable via HR and HRV and that these changes in cardiac function will alter systemic blood flow and be reflected in PR and PRV metrics. Therefore, in an effort to measure stress responses reflected in autonomic changes that influence cardiac activity, ECG measures these phenomena more proximally than PPG, while PPG captures more downstream physiologic variables that are vulnerable to more physiologic interference and require reliance on a greater number of assumptions (eg, effects of the respiratory cycle on blood flow and multifactorial variance in systemic vascular resistance, [26] as a well-known, extreme example of uncoupling between ECG and PPG within this conceptual chain, consider the pathological state of pulseless electrical activity). The adequacy of PRV as a surrogate for HRV remains controversial [26-31], and to the knowledge of this author’s group, no studies have been published to evaluate the suitability of PRV analysis to study physician wellness.

This study aimed to evaluate the feasibility of collecting and analyzing PPG data from EPs while they work and to investigate whether patterns in their PR and PRV changed over the course of an academic year. To achieve this aim, PPG data collected from resident and attending EM physicians during the fourth quarter of the year were compared with an analogous PPG dataset from the same providers during the first quarter of the same year. This approach is founded on the notion that as trainees gain experience in their role over the course of a year, perception of stressors and their physiologic manifestations among the trainees—as well as among the attending physicians directly responsible for them—should decrease. For example, it has been shown that the odds that an individual EP will register a detectable concentration of salivary cortisol after completing a shift significantly decrease over the course of a year [32]. An additional analysis will be restricted to the resident-only subgroup to investigate whether patterns in PR and PRV may be specific to EM trainees. Given the novelty of the methods employed in this pilot study, a secondary aim involved assessing whether currently available PPG technology is ready for application to the ecologically valid study of physician wellness (ie, real-time examination of autonomic manifestations of stress at work), embedded within the context of a comprehensive literature review. In summary, the essential purpose of this pilot study is to answer the question: Can wearable PPG sensors offer a scalable, objective, and ecologically valid method to study workplace stress among EPs?

## Methods

### Institutional Review

This study was approved by the Institution’s Committee on Clinical Investigations and the Department of Emergency Medicine’s Medical Education Executive Committee.

### Study Setting and Population

Participants were recruited from an academic ED in late June 2016 and 2017 at the beginning of the academic year to support 2 iterations of the study in consecutive years. Following an introductory information session during a scheduled department conference, 3 emails were sent to all EM residents and attending physicians inviting them to participate in the study. Volunteers who responded to these recruitment emails underwent an informed consent procedure, individually conducted by a trained research assistant. No financial compensation was offered for participation. All recordings took place at a single site: the ED at the host academic medical center.

### Data Collection

Participants wore an Empatica E4 PPG biosensor watch (Empatica Inc) during at least 1 shift in the ED at the host institution during the first quarter (Q1: July-September) and final quarter (Q4: April-June) of 1 academic year. Continuous PPG data were recorded over the course of the entire 9-hour shift (14:00-23:00) while performing routine clinical care. Each recording automatically generated 2 forms of data used for this study: a raw PPG signal (BVP data) and an interbeat interval (IBI) log, each measured with respect to time at a sampling frequency of 64 Hz. Compilation of these datasets relied upon standard detection and filtering algorithms developed by Empatica [33,34].

### Data Filtering and Inclusion Criteria

**Figure 1:**
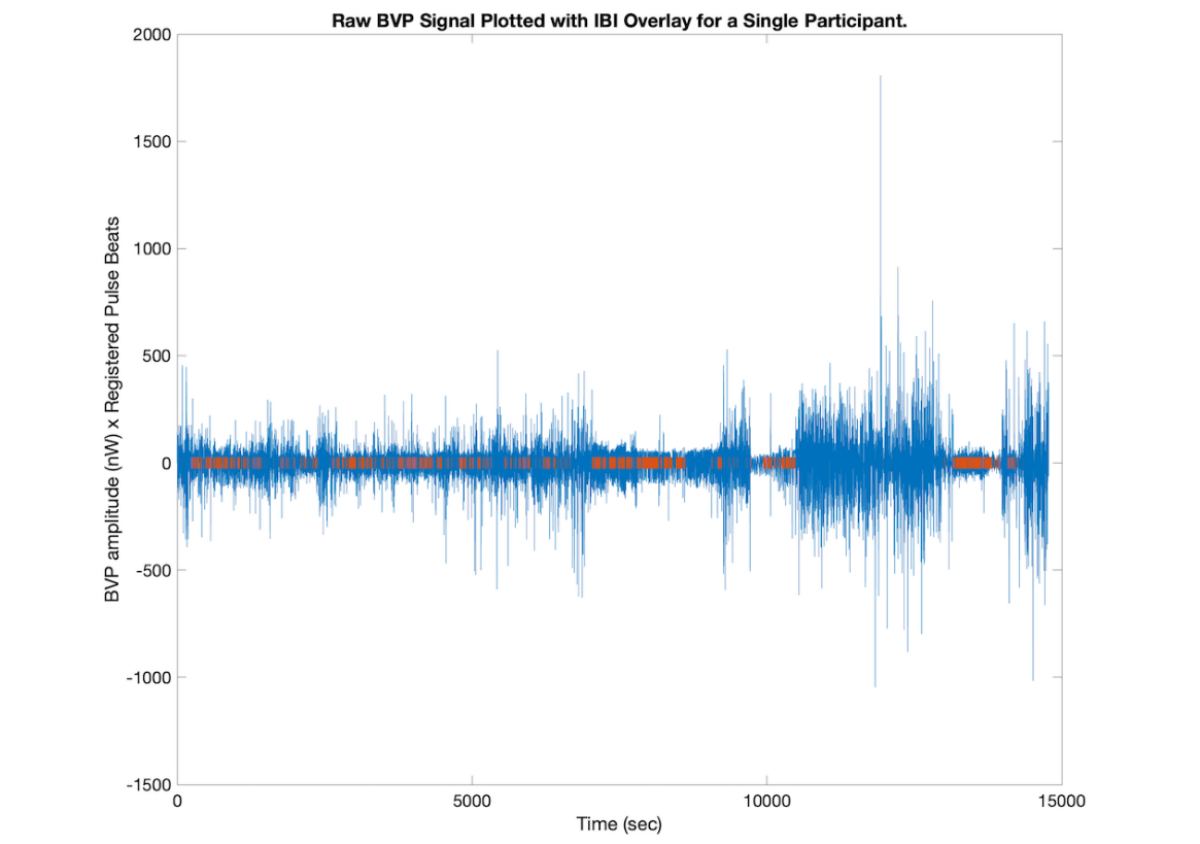
Raw blood volume pulse (BVP) amplitude measured in nanowatts from a single PPG recording plotted in blue, with registered pulse beats from the corresponding interbeat interval (IBI) log marked in red. PPG: photoplethysmography.

Once compiled, each recording entered a screening process before being included in the study. Data filtering and analysis procedures were executed via a self-developed set of MATLAB scripts designed to accomplish the following operations (Mathworks). First, the timing of each PPG recording was compared with the timing of each shift to verify that each included dataset reflects the participant’s physiology while providing care in the ED. Second, the total number of heart beats registered in each IBI dataset was screened for a minimum total of 300 beats. Given that each IBI dataset is generated via the application of a standard heart beat detection algorithm that excludes segments of recording consistent with artifacts or low signal to noise ratio, this screening measure served to ensure sufficient clean signal density in each recording. Third, each recording that met these inclusion criteria was plotted in a PPG versus time graph, and additional markers were superimposed on the x-axis to indicate time periods included in the IBI logs (Figure 1).

These plots were visually inspected to add a final opportunity to manually detect segments of included IBI data sourced from unreliable PPG signal. Finally, to sustain a repeated-measures analysis, data were only included from participants with 1 satisfactory recording in both Q1 and Q4. Once included, each PPG and IBI dataset was entered into 2 primary analyses, one in the time domain and the other in the frequency domain, providing complementary assessments of PR and PRV over time on a repeated-measures basis.

### Data Analysis

Each IBI dataset was entered into a time-domain analysis designed according to previously validated standards [35]. Variables of interest included median PR, mean R-R interval, standard deviation of R-R interval, and root mean square of successive difference of R-R interval (RMSSD). Calculations of RMSSD were performed by isolating the single longest stretch of consecutive heart beats detected within each IBI log. The PPG dataset yielded calculations of power in the low-frequency range (LF: 0.05-0.15 Hz) and high-frequency range (HF: 0.15-0.40 Hz), as well as LF:HF ratio (LHR), composing the frequency-domain analysis [35].

Recordings dated from Q1 and Q4 were separated into 2 time points for each participant. The previously described standard measures of PR and PRV were calculated for each recording, and Q1 versus. Q4 was compared via paired Student *t* tests for each metric. The data used in this study satisfy the requisite assumptions upon which paired-samples *t* tests rely. Statistical power for these tests was calculated using a publicly available MATLAB algorithm [36].

Toward the aim to assess the quality of the data collected through these methods, the IBI log from each of the original recordings included in the analysis was transformed into a time vector that only included 1-second segments of time during which pulse beats were detected (including the interval between consecutive beats), according to the standard Empatica algorithm. This vector was then transposed upon a second vector representing the total recording time captured in each corresponding PPG dataset, sampled at the same frequency (graphically represented in Figure 1). Finally, a quotient of time during which pulse beats were detected over the grand total recording time, in seconds, was computed to determine the overall percent of data that was ultimately interpretable with respect to time.

## Results

A total of 51 EPs contributed 95 recordings, producing over 800 hours of data. Following application of the previously described screening procedure, roughly 440 hours of data from 21 EPs were included in the study (Table 1). Interpretable PPG data constituted 8.54% of the total recording time undertaken in this study. A total of 10 participants were trainees and 11 were attending physicians. Two participants were included in both years of the study. Five of the 21 participants were female, including 2 of the 10 residents. Residents from all years of training were represented, including 4 postgraduate year (PGY)-1, 2 PGY-2, and 4 PGY-3 trainees. The mean age of participants at the time of enrollment was 32.8 years, with 29.9 years and 35.6 years for residents and attending physicians, respectively.

**Table:** Descriptive characteristics of participants included in the study.

Characteristic		Value
**Sample size (N=21)**		
	Resident physicians, n (%)	10 (48)
	Attending physicians, n (%)	11 (52)
**Sex (female), n (%)**		
	Overall sample (N=21)	5 (24)
	Resident physicians (n=10)	2 (20)
	Attending physicians (n=11)	3 (27)
**Age (years), mean (SD)**		
	Overall sanmple (N=21)	32.8 (5.9)
	Resident physicians (n=10)	29.9 (3.9)
	Attending physicians (n=11)	35.9 (6.3)
**Trainees**		
	Total	10 (100)
	PGY^a^-1, n (%)	4 (40)
	PGY-2, n (%)	2 (20)
	PGY-3, n (%)	4 (40)

^a^PGY: postgraduate year.

Time-domain analysis revealed no significant changes in PR or PRV in Q4 compared with Q1. Table 2 details the descriptive data from the primary analysis. Median PR in Q4 was 75.8 (SD 13.5) compared with 76.8 (SD 10.8) in Q1 (*P*=.57). Comparisons of PRV measures between Q4 and Q1 also did not reach statistical significance, including group mean RMSSD of 0.81 (SD 0.16) versus 0.80 (SD 0.11), respectively (*P*=.96). Similarly, frequency-domain analysis did not show any differences in Q4 versus Q1, including a comparison of LHR in which group means were 0.42 (SD 0.04) and 0.41 (SD 0.03), respectively (*P*=.43). These negative findings were observed despite adequate power, with estimated power (1−ß) values exceeding .90 for each of the 8 Student *t* tests included in this study, corresponding to the 8 PR and PRV measures of interest used, as outlined in Table 3.

**Table 2:** Mean (SD) values of measures in primary analysis including all participants (XeY denotes scientific notation).

Quarter	MPR^a^	MRRI^b^	SDNN^c^	RMSSD^d^	LF^e^	HF^f^	LHR^g^
Quarter 1	76.8 (10.8)	0.80 (0.113)	0.11 (0.035)	0.80 (0.113)	3.4e+3 (1.7e+3)	8.3e+3 (4.2e+3)	0.41 (0.04)
Quarter 4	75.8 (13.5)	0.81 (0.137)	0.11 (0.023)	0.81 (0.161)	3.5e+3 (2.1e+3)	8.5e+3 (5.3e+3)	0.42 (0.03)

^a^MPR: median pulse rate (pulse beats per minute).

^b^MRRI: mean R-R interval (seconds per pulse beat).

^c^SDNN: standard deviation of R-R interval.

^d^RMSSD: root mean square of successive difference of R-R interval.

^e^LF: low-frequency power (ms^2^).

^f^HF: high-frequency power (ms^2^).

^g^LHR: ratio of LF to HF.

**Table 3:** Results of primary analysis including all participants (Student t tests).

Metric	MPR^a^	MRRI^b^	SDNN^c^	RMSSD^d^	LF^e^	HF^f^	LHR^g^
*P* value	.57	.32	.93	.96	.79	.91	.43
Power (1−ß)	.94	.92	.95	.95	.94	.95	.90

^a^MPR: median pulse rate (pulse beats per minute).

^b^MRRI: mean R-R interval (seconds per pulse beat).

^c^SDNN: standard deviation of R-R interval.

^d^RMSSD: root mean square of successive difference of R-R interval.

^e^LF: low-frequency power (ms^2^).

^f^HF: high-frequency power (ms^2^).

^g^LHR: ratio of LF to HF.

A secondary analysis of the resident-only subgroup similarly failed to reveal any significant changes in PR or PRV in Q4 compared with Q1, despite estimated power values greater than .80. Tables 4 and 5 detail results from this secondary analysis.

**Table 4:** Mean (SD) values of measures in primary analysis including trainees only (XeY denotes scientific notation).

Quarter	MPR^a^	MRRI^b^	SDNN^c^	RMSSD^d^	LF^e^	HF^f^	LHR^g^
Quarter 1	78.5 (10.5)	0.78 (0.095)	0.12 (0.04)	0.79 (0.123)	3.6e+3 (1.7e+3)	8.8e+3 (4.2e+3)	0.41 (0.02)
Quarter 4	74.7 (12.5)	0.82 (0.123)	0.11 (0.022)	0.82 (0.119)	3.6e+3 (2.5e+3)	8.7e+3 (6.2e+3)	0.42 (0.02)

^a^MPR: median pulse rate (pulse beats per minute).

^b^MRRI: mean R-R interval (seconds per pulse beat).

^c^SDNN: standard deviation of R-R interval.

^d^RMSSD: root mean square of successive difference of R-R interval.

^e^LF: low-frequency power (ms^2^).

^f^HF: high-frequency power (ms^2^).

^g^LHR: ratio of LF to HF.

**Table 5:** Results of secondary analysis including trainees only (Student t tests).

Metric	MPR^a^	MRRI^b^	SDNN^c^	RMSSD^d^	LF^e^	HF^f^	LHR^g^
*P* value	.28	.15	.44	.49	.99	.95	.16
Power (1−ß)	.88	.83	.93	.92	.95	.95	.81

^a^MPR: median pulse rate (pulse beats per minute).

^b^MRRI: mean R-R interval (seconds per pulse beat).

^c^SDNN: standard deviation of R-R interval.

^d^RMSSD: root mean square of successive difference of R-R interval.

^e^LF: low-frequency power (ms^2^).

^f^HF: high-frequency power (ms^2^).

^g^LHR: ratio of LF to HF.

A post hoc analysis of the original 95 recordings demonstrated a yield of 8.54% with respect to time, meaning less than one-tenth of the PPG data recorded contained useful substrate for the PR and PRV analyses employed in this study.

## Discussion

### Principal Findings

This study demonstrates the feasibility of recording real-time physiological data in EPs while they practice in an operational sense but fails to support the notion that the current state of wearable PPG biosensor technology can efficiently and reliably measure variables of interest in a meaningful way. The evidence produced by this study consistently supports the null hypothesis that there is no difference in PR or PRV patterns among EPs at the end of an academic year compared with the beginning, or more precisely, fails to support the alternative hypothesis that such a difference exists. The primary analysis failed to detect any differences over time in EPs at large, and the secondary analysis demonstrated a similar inability to find any significant changes among EM residents. These negative findings were observed in both arms of the study despite the use of complementary time-domain and frequency-domain analyses and despite adequate power calculated for each analysis. Given the anticipated effect of the passing academic year to decrease physiologic stress—including attending physicians working with more experienced and autonomous trainees, and particularly for residents with the benefit of 6 to 10 months of training in their assigned role—the question of whether this methodology can capture such effects requires careful consideration. Therefore, the aim of this pilot study to assess the suitability of these methods for these purposes emerges as paramount.

The closest precedent for this study is a 1998 study that used ECG recordings in 12 EPs to report increased sympathetic tone during night shifts [37]. Clearly, additional research is needed to develop objective, ecologically valid metrics of wellness among EPs, ideally using tools that can be upscaled to make widespread participation possible even while providing care in the ED. As previously mentioned, no literature exists on the use of PPG to study physician wellness—in any specialty—to the knowledge of this author’s group. However, a wealth of research continues to focus on evaluating the adequacy of PPG technology to approximate the HRV analyses that have demonstrated promise in this field [13-15]. This body of research comparing the 2 methodologies has yielded mixed results. The literature seems to weigh in favor of the accuracy of PPG measurement for healthy human subjects at rest [27]. On the other hand, evidence consistently calls into question the ability of PPG-enabled wearable biosensors to achieve useful recordings in human subjects undergoing active tasks marked by interference from factors such as motion artefact, inconsistent skin contact, and increased degrees of respiratory-cardiovascular interaction among other sources of physiologic variability [26,28,38]. Of course, these conditions apply to the study of EPs providing care in the ED and likely account for the yield of less than 10% usable data from our total recording time. In addition, further ambiguity surrounds more specific applications of HRV concepts to PRV methodology, such as translation of frequency domain metrics from ECG to PPG data [29,30], and even more fundamentally, use of the frequency domain to assess sympathetic activity in either ECG or PPG [20-23].

Taking this body of the literature into consideration, this study adds several new pieces of information. The primary goal of this study was to investigate whether any patterns in PR and PRV among EPs could be detected by comparing PPG data collected during Q4 versus Q1 of an academic year. These results indicate that no such patterns were found, regardless of the domain of analysis or training status of participants included and despite adequate power to conduct each test. The secondary question that emerges from this conclusion is whether the novel methodology of this study possesses the ability to detect changes in physiology linked to stress if they do in fact exist.

On the basis of a focused review of the pertinent literature, it seems unclear at best whether wearable PPG sensor technology has yet been developed to the extent necessary to characterize stress levels among EPs, especially while providing care in the ED. In particular, the quality assessment analysis demonstrating a yield of useful data less than 10% suggests that this methodology is at least an inefficient, if not inaccurate, means of probing physiologic changes in EPs at work. Taken together, the results of this study and existing body of the literature seem to suggest that PPG technology has not yet matured to the degree required for the use of PRV analysis in this field of research. However, it remains crucial to understand that wearable PPG sensors lie at the center of an intensely active area of multidisciplinary research and technological development [39]. For example, a new singular spectrum analysis–based method to automate detection and correction of artefacts in ECG data was published in 2019 following the completion of this study; such advancements might significantly enhance the quality of data that is readily accessible to researchers without a background in the technical aspects of this field [40]. Although this study suggests that current PPG-enabled wearable biosensors technology might have limited utility in the study of physician wellness in real time, advancements in the collection, analysis, and interpretation of PPG data will likely transform the field in the near future and warrant further investigation.

### Limitations

Consideration of future developments surrounding this methodology aside, further research in the short term is needed to study sources of burnout in EPs. For example, several limitations of this study will need to be addressed in future investigations. First, as previously discussed, the current state of wearable PPG sensor technology remains highly vulnerable to interference from settings such as the ED. Perhaps, studies of smaller scope using HRV must be used to investigate EP wellness until PRV analysis has reached requisite maturity for these purposes. Alternatively, perhaps a chest strap can be used as an intermediate wearable biosensor in future studies to reduce interference inherent to PPG measurement at the wrist without subjecting participants to mobile ECG recording. Second, although the repeated-measures design of this study was chosen to enhance sensitivity to detect changes within participants over time, it unfortunately limited sample size, especially among trainees. Resident EM physicians at the host academic hospital frequently rotate at outside ED locations, significantly limiting opportunities to capture recordings within both time windows. Perhaps, an alternative design with greater sample size might uncover significant patterns in PRV among EPs, although the power calculations performed in this study call such considerations into question. Third, in an effort to test whether this methodology can offer a time-sensitive, ecologically valid way to study EPs at work, the exact duration of recordings entered into these analyses was not precisely standardized, which can pose issues regarding the frequency-domain analyses in particular. Instead, participants were simply instructed to record PPG throughout their entire 9-hour shift, and each recording that met inclusion criteria as detailed in the Methods section was entered into the frequency-domain analysis in its entirety. Similarly, the devices used in this study are capable of recording accelerometer data that can be used to inform exclusion of PPG data associated with significant motion. This measure was not taken for the purpose of this study because of the concern that exclusion of times at which EPs are active might lead to the systematic loss of the stressors this pilot study aimed to capture, but future studies of this kind must carefully weigh the benefits and disadvantages of this decision with respect to the primary goals of the study.

Fourth, this study lacked a direct comparison between HRV and PRV. The primary goal of this study was to evaluate a novel tool to measure stress among EPs in real time to guide efforts aimed at alleviating physician burnout. As a result, the focus of this study was to assess the utility of PRV analysis, given its readiness for widespread application to resident and attending EM physicians, rather than to validate the technology used for these purposes against another that bears limited ability to serve this primary goal. However, further research is recommended to use ECG alongside PPG to compare HRV versus PRV analyses in EPs providing care in the ED. Such studies can more directly assess the still unclear question as to whether the physiologic changes investigated in this study exist but cannot be detected by PRV analysis versus the possibility that these changes simply do not exist. Similarly, this study did not use self-reported data as a comparator, although evidence suggests that caution must be used when relying upon the assumption that different modalities of multifactorial concepts such as stress should necessarily correlate with one another [17]. Future studies will be required to address these limitations and further advance the search for new objective tools that can characterize burnout among EPs.

### Conclusions

The results of this study do not indicate any changes in PR or PRV among EPs over the course of an academic year. Due to the limitations of this study, it remains unclear whether such patterns exist and merely went undetected, although calculations suggest this study was sufficiently powered. The finding that less than one-tenth of the recording time dedicated to this study yielded useful substrate for PR and PRV analyses lends support to the notion that PPG technology might not yet be ready for application to these purposes, suggesting that the methods used in this study are poorly suited to test the hypothesis in question. A review of the literature supports the suggestion from this study that wearable PPG sensor technology has not yet matured to the extent required for accurate measurement of physiologic reflection of mental stress among EPs while at work on a large scale. However, active research and development surrounding this technology will likely offer new opportunities for investigation in the near future. Further research is required to identify new tools that can inform mounting efforts to alleviate physician burnout.

In summary, this study adds the first evaluation of wearable PPG biosensor technology as an ecologically valid, objective measure of workplace-related stress among EPs to the literature. Although this methodology proved to be low-burden and therefore easily scalable, data quality was a prohibitive issue. At the least, the current state of wearable PPG biosensors is subject to technological limitations that render it unable to reliably measure PR and PRV in active EPs. At the most, PPG methodology is subject to physiologic interference that precludes study of upstream concepts such as sympathovagal balance as a reflection of stress among physically active participants, such as EPs during shifts. In conclusion, this pilot study suggests that alternative methods must be explored to establish an objective, scalable, ecologically valid way to measure stress among EPs at work.

## Abbreviations

BVP: blood volume pulse
ECG: electrocardiography
EM: emergency medicine
EP: emergency physicians
HF: high frequency
HR: heart rate
HRV: heart rate variability
IBI: interbeat interval
LF: low frequency
LHR: ratio of LF to HF
MPR: median pulse rate (pulse beats per minute)
MRRI: mean R-R interval (seconds per pulse beat)
PGY: postgraduate year
PPG: photoplethysmography
PR: pulse rate
PRV: pulse rate variability
RMSSD: root mean square of successive difference of R-R interval
SDNN: standard deviation of R-R interval
